# Iron Oxide–Modified Carbon Electrode and Sulfate-Reducing Bacteria for Simultaneous Enhanced Electricity Generation and Tannery Wastewater Treatment

**DOI:** 10.3389/fbioe.2021.747434

**Published:** 2021-11-12

**Authors:** Faiz Miran, Muhammad Waseem Mumtaz, Hamid Mukhtar, Sadia Akram

**Affiliations:** ^1^ Department of Chemistry, University of Gujrat, Gujrat, Pakistan; ^2^ Institute of Industrial Biotechnology, Government College University, Lahore, Pakistan

**Keywords:** microbial fuel cell, tannery wastewater treatment, sulfate-reducing bacteria, iron nanoparticles (Fe NPs), electricity generation

## Abstract

The microbial fuel cell (MFC) is emerging as a potential technology for extracting energy from wastes/wastewater while they are treated. The major hindrance in MFC commercialization is lower power generation due to the sluggish transfer of electrons from the biocatalyst (bacteria) to the anode surface and inefficient microbial consortia for treating real complex wastewater. To overcome these concerns, a traditional carbon felt (CF) electrode modification was carried out by iron oxide (Fe_3_O_4_) nanoparticles via facile dip-and-dry methods, and mixed sulfate-reducing bacteria (SRBs) were utilized as efficient microbial consortia. In the modified CF electrode with SRBs, a considerable improvement in the bioelectrochemical operation was observed, where the power density (309 ± 13 mW/m^2^) was 1.86 times higher than bare CF with SRBs (166 ± 11 mW/m^2^), suggesting better bioelectrochemical performance of an SRB-enriched Fe_3_O_4_@CF anode in the MFC. This superior activity can be assigned to the lower charge transfer resistance, higher conductance, and increased number of catalytic sites of the Fe_3_O_4_@CF electrode. The SRB-enriched Fe_3_O_4_@CF anode also assists in enhancing MFC performance in terms of COD removal (>75%), indicating efficient biodegradability of tannery wastewater and a higher electron transfer rate from SRBs to the conductive anode. These findings demonstrate that a combination of the favorable properties of nanocomposites such as Fe_3_O_4_@CF anodes and efficient microbes for treating complex wastes can encourage new directions for renewable energy–related applications.

## Introduction

The current challenges faced by the world regarding sustainable energy production and water pollution require the development of novel technologies that can aim to amicably solve these problems (Luderer et al., 2019; Sun et al., 2020). The microbial fuel cell (MFC) is developing as a potential biotechnology which can help in tackling these challenges as electricity production and wastewater treatment are concurrently accomplished by bacteria as biocatalysts (Yaqoob et al., 2020; Yaqoob et al., 2021). Importantly, such a technology aiming at energy self-sufficiency in wastewater treatment systems is highly vital for the developing countries which often lack the fiscal capitals required for the proper wastewater treatment infrastructures (Gu et al., 2017; Maktabifard et al., 2018). Given that lower MFC efficiencies are a hurdle in its commercialization, and the anode electrode and microbial communities play a fundamental role in electricity generation and pollutant removal (Choudhury et al., 2017), research on these aspects can help in enhancing the performance of MFCs to commercially acceptable levels.

Tanneries are water-intensive industries which use as much as 70 L of water in processing 1 kg of raw hide/skin to manufacture leather (Thanikaivelan et al., 2004), where downstream effluents become toxic with chemicals and organic impurities (Rafique et al., 2010). Tanneries also produce sulfate-rich wastewater owing to the use of H_2_SO_4_ and the addition of sulfides in the unhairing process that oxidizes to sulfate before entering into the sewer (Galiana-Aleixandre et al., 2011). Since polluted water with a large number of noxious contaminants discharged from these tanneries penetrates the water tables, it causes severe diseases of the respiratory tract and digestive system to humans (Bhalli and Khan, 2006). Therefore, treating tannery waste before discharging into the water bodies is of great importance. The biological treatment of tannery wastes in comparison to physical, chemical, and advanced oxidation approaches is an inexpensive and green approach with lower sludge generation (Durai and Rajasimman, 2011). MFCs can aid in further accelerating the biodegradation of tannery pollutants, as the MFC has an infinite terminal electron acceptor (anode) in comparison to exhaustible electron acceptors like O_2_ and NO_3_^−^, with electricity production as an added benefit (Chen et al., 2016). To this end, local tannery wastewater from Sialkot (an industrial city), Pakistan, was evaluated for treatment in the MFC system. Currently, more than 270 tanneries are operating in about ten clusters in the vicinity of Sialkot and accommodate the increasing manufacturing needs. Sulfate was first reduced to sulfite and then reoxidized to sulfate by forming thiosulfate as an important intermediate or by direct sulfite oxidation.

The microbial consortia of the MFC can considerably affect the electricity production capacity and treatment efficiency (Choudhury et al., 2017; Hassan et al., 2018). Incidentally, the sulfate-reducing bacteria (SRBs) have been found to be a very potent group for efficient generation of current as compared to the electroactive environmental bacteria already known (i.e., *Geobacter* and *Shewanella*) (Kang et al., 2014). Zhang et al. (2009) have shown the probability of manipulating the SRBs for transforming sulfates to sulfides, and then electrons can be obtained indirectly via abiotic oxidation of microbially generated sulfide at the anode. In another study, Lee et al. (2012) have proven that the SRB group can transform sulfates to sulfides, and then the MFC anodic biofilm can carry out the oxidation of sulfide to elemental sulfur along with electricity generation. Redox voltage of the half-cell also plays a significant role in such systems. For instance, partial sulfur reuse in electrode potential control MFCs has also been shown to participate in current generation. At lower poised potentials, thiosulfate was detected as the reduced product of sulfate and was effectively oxidized, particularly under -0.1 V vs SHE. The anode at lower poised potentials, for example, -0.2 V, largely accumulates genes for thiosulfate and sulfite reduction. The oxidation of thiosulfate to tetrathionate and sulfide to sulfur or polysulfide has also been confirmed at lower poised potentials of -0.1 and -0.2 V vs SHE (Zhang et al., 2020a). It has also been revealed that efficient removal of sulfate and organic carbon by the MFC is possible with the help of the anodic biofilm of SRBs and sulfide-oxidizing bacteria (Lee et al., 2014). These previous investigations revealed only the potential of SRBs for simple carbon source biodegradation, sulfate reduction, and electricity production; however, the advanced capabilities of SRBs in MFCs are still unexplored. SRB comprise a diverse group of essential anaerobes, which have the capacity of dissimilating sulfate to sulfide and to oxidize many types of substrates (Willis et al., 1997). Based on the potential of SRBs, huge importance is being given for the implementation of SRBs for the complex industrial waste remediation. For example, SRBs have been utilized for the biotreatment of many complex contaminants such as phenanthrene, cyclohexane, naphthalene, metals, and 2,4,6-trinitrotoluene (Hussain et al., 2016). Furthermore, SRB groups can also attain extraordinary removal of complex organic waste and sulfate under anaerobic environments (Rasool et al., 2013). Consequently, SRBs would be a potent microbial culture in MFCs, which might enhance the overall waste product treatment capability and energy generation from tannery wastewater.

Electroconductive nanoparticles have been shown to transfer electrons across the outer membrane (OM) of Gram-negative microbes. Metallic nanoparticles synthetically produced on the surface of the cell-aided electron transport from the inside of the cell across the OM to the cell outer surface, thus functioning as electron conductors across the OM (Malvankar et al., 2015). Likewise, it has been shown that the biosynthesized iron-based nanocluster attachment with the cell surface provides a pathway for electron transfer across the OM to extracellular electrodes (Nakamura et al., 2010; Zhou et al., 2014). Furthermore, the model of electron transport to electrodes by the SRBs has been suggested (Murugan et al., 2018). Thus, the possibility of iron-based nanocluster-mediated electron transport to the anode via SRB has offered new ways to enhance the efficiency of SRB MFCs. Since this has not been explored before in MFCs for real wastewater systems, we are combining the two strategies to improve MFC efficiency.

The aim of this study was to evaluate the MFC performance utilizing a carbon felt electrode fabricated with Fe_3_O_4_ nanoparticles for the treatment of tannery wastewater by applying mixed SRB in an anaerobic chamber of the MFC, with simultaneous bioelectricity generation. Many characterization techniques such as FTIR, XRD, and TEM were employed to confirm the successful synthesis of Fe_3_O_4_ nanoparticles. The bioelectrochemical performance of the MFC was estimated by the generation of voltage, power, and current density. Furthermore, wastewater treatment efficacy was assessed by the removal of chemical oxygen demand (COD) and sulfate reduction. Although continuous flow systems have many advantages for studying fuel cells (Habermann and Pommer, 1991), here we used the batch MFC system for making it less complicated and easier to establish and manage for our first such study where we are treating complex wastewater using an anode-modified electrode and mixed electroactive SRBs.

## Materials and Methods

### Preparation of Fe_3_O_4_ Nanoparticles

Fe_3_O_4_ nanoparticle synthesis was carried out by adopting a solvothermal method, as previously reported along with some moderations in the procedure (Zhang et al., 2020b). 1 g of FeCl_3_.6H_2_O was added to 20 ml of ethylene glycol, and a clear solution was obtained upon stirring. After that in the acquired solution, 10 ml of ethylene diamine and 3 g of sodium acetate were added and stirred at ambient temperature for 30 min. The mixture was then poured into a Teflon-lined autoclave having 50 ml capacity and was heated in an oven at 180°C for 8 h. The resultant black-colored product was washed repeatedly with distilled water/ethanol, followed by desiccation to gain a dry product. This drying led to the formation of a magnetic iron powder.

### Characterization of Fe_3_O_4_ Nanoparticles

The formation of Fe_3_O_4_ nanoparticles, their purity, size, and morphology were affirmed by the help of various analytical techniques. Dimensions, crystal phase, and size of the nanoparticles were evaluated by X-ray diffraction, using an X’pertpro (PANalyatical) XRD model. FT-IR spectroscopy of the nanoparticles in the scanning range 4,000–600 cm^−1^, using the Cary 630 Agilent FT-IR spectrometer, was performed to check the purity of the nanoparticles. Morphological characterization was carried out by transmission electron microscopy (TEM) (Tecnai G^2^ F20 U-TWIN), with an accelerating voltage of 200 kV. The size and morphology of the nanoparticles were also assessed by SEM microscopy (Model JSM5910, JEOL, Japan), with 30kV energy, a maximum magnification of 300,000x, and 2.3 nm resolving power, while the purity and elemental ratio of iron and oxygen in nanoparticles were estimated by using EDX (JSM5910) (INCA200/Oxford instruments, United Kingdom).

### Preparation of Fe_3_O_4_@CF Electrode

The coating of nanoparticles on the surface of electrodes was effectively achieved by dipping CF anodes in a Teflon-based autoclave in the solution of Fe_3_O_4_ sustained at 180°C for 20 h. The autoclave was cooled down to room temperature (25°C), and deionized water was used to wash the Fe_3_O_4_-coated anodes. The surface-modified electrodes were then placed in the vacuum oven at 60°C for 24 h for the purpose of drying. The amount of Fe_3_O_4_ deposited on the surface of Fe_3_O_4_@CF was measured as a count of difference in the weight of electrodes before and after the process of coating that was estimated to be 8.3 mg, suggesting that CF exhibited high potential for Fe_3_O_4_ coating by this methodology.

### Tannery Wastewater and Microbial Culture

Tannery wastewater was obtained from local tanneries in the tannery zone, Sialkot, Pakistan, in 1-L glass bottles, which was mixed in a large 5-L beaker to get the representative sample. The samples were stored at 4 C before the physicochemical characterization and used in the MFC as the anolyte. Physicochemical compositions of the tannery wastewater are provided in Table 1. The initial microbial inoculum was also collected from a tannery wastewater treatment plant in the tannery zone, Sialkot, Pakistan. The collected sludge was pretreated by removing the foreign particles (metals) by sieving, and the final sludge obtained was stirred at 150 rpm for 1 h to obtain uniform sludge in the liquid phase. Afterward,the processed sludge was introduced into two 1-L Erlenmeyer flasks sealed with stoppers.

**TABLE 1:** Physicochemical composition of tannery wastewater.

Parameter	Value
TDS (g/L)	4.450
TSS (g/L)	0.3345
COD (g/L)	3.455
BOD (g/L)	0.224
Cr (mg/L)	6.44
Ni (mg/L)	0.22
Mn (mg/L)	0.17
Cd (mg/L)	0.74
EC dS/m	17.92

### Selective Enrichment of SRB

Selective enrichment of the active SRB cultures was attained by Postgate B medium (Postgate, 1984) that used lactate as the electron donor and carbon source. The main components of the medium were given as follows (in g/L): sodium lactate (3.5); K_2_HPO_4_ (0.5), NH_4_Cl (1.0), FeSO_4_.7H_2_O (0.5), MgSO_4_.7H_2_O (2.0), CaSO_4_ (1.0), yeast extract (1.0), ascorbic acid (0.1), and thioglycolic acid (0.1). The culture was also supplemented with bromoethanesulfonic acid (BESA) (3.2 g/L) during the enrichment phase for inhibiting the methanogens that can consume the lactate medium. The pH of the growth medium at the start was kept at 7.5 ± 0.1 using 1 N NaOH or HCl solution. Furthermore, the sterilization of the medium was carried out for 20 min at 15 psi and 120°C. Anaerobic conditions were maintained by purging N_2_ gas in the medium before inoculation. 1-L bottles were used for enrichment which were kept inside the incubator and were shaken using a rotary shaker at 120 rpm and 30°C. The enrichment was confirmed by the reduction of sulfate to sulfide, signified by change in the media color to black. The enrichment of SRB culture was further enhanced by subculturing every week using a fresh medium after decanting 70% of the supernatant from the culture bottle. After a period of 4 weeks, a culture potentially having a dense population of mixed SRBs was achieved. This enriched SRB culture was then used in MFCs. A non-enriched culture was used as the control.

### Assembly and Operation of the MFC

Two-chamber MFC systems were employed in this research. Each anodic and cathodic chamber had a working volumetric capacity of 200 mL. The anode used in the experimental work was 5-cm × 5-cm carbon felt (CF) (Alfa Aesar, United States) with 3.18 mm thickness or CF coated with Fe_3_O_4_, while platinum-fabricated carbon cloth was utilized as the cathode (1.00 mg cm^−2^ 20 wt% Pt (5 × 5 cm); Fuel Cell Earth, Wakefield, United States). A minimum separation was ensured between the electrodes for reducing the internal resistance (R_int_) using a proton (H^+^) exchange membrane (Nafion^®^ 117, Dupont Co., United States). The proton exchange membrane was treated with H_2_O_2_, sulfuric acid, and deionized water to improve MFC performance. The external resistor with an external resistance (R_ext_) of 500 Ω was used to complete the circuit by linking the electrodes. The temperature of the MFC system was sustained at 30°C using a water bath. The anolyte medium of tannery wastewater was diluted to the COD levels of 500 mg/L and COD/SO_4_^2−^ of 2.0 by using nutrients with compositions as follows: NaHCO_3_, 420 mg L^−1^; CaCl_2_, 15 mg L^−1^; and 1 ml of trace mineral solution. (NH_4_)_2_SO_4_, MgSO_4_.7H_2_O, and MnSO_4_·H_2_O were also added as nutrients to maintain consistent initial sulfate concentration. The MFC medium was sparged with nitrogen. A magnetic stirrer was used in the anodic chamber for stirring, and anaerobic conditions of the anodic chamber were maintained by purging N_2_ gas. In the cathodic chamber, 0.1 M phosphate buffer with pH 7.0 was added as the catholyte, and O_2_ (from air) was delivered via an air pump equipped with a regulator. Ports were provided to each chamber for feeding, withdrawing samples, and withdrawing the ultimate treated medium. For the MFC setup, 20% sludge and 80% anolyte mixture were used to sustain the growth of the biofilm on the anodic electrode; 50% medium was decanted for the first three runs and superseded with sludge and fresh medium with the same ratio (1:4). After the third cycle, only the anolyte was added for subsequent cycles.

### Estimation of Electrochemical Performance

The voltage was measured using a multimeter that was attached across the anodic and cathodic electrodes, and voltage data were monitored at regular intervals. The logged voltage (V) was utilized to calculate the current (I) using Ohm’s law (I = V/R). The power density (P), standardized to the anodic surface area facing the anodic chamber, was measured using the equation P = V^2^/(R_ext_.A), where A (m^2^) = anodic surface area, R_ext_ (Ω) = the external circuit resistance, and V [Volts] = MFC potential. By changing the R_ext_ step by step from 10 KΩ to 10 Ω, the polarization curve was acquired. Every resistor was retained for 30 min, and the voltage was stable.

COD (chemical oxygen demand) was calculated by the standard method reported previously (Greenberg et al., 1992). Percentage removal of COD was acquired by the following relation:

$$COD\;removal\;\%\;=\;\frac{\;\left(COD_{0}-COD_{f}\right)}{COD_{f}},$$

where COD_*0*_ and COD_*f*_ represent the COD measured at time t = *0* and final time t = f, respectively. Sulfates were measured using the standard APHA method.

The Coulombic efficiency (CE) of the MFC was estimated by the relation given as follows:

$$CE\%=\frac{8\times I\times \Delta t}{\Delta COD\times V_{\mathrm{an}}\times 96,485}\times \;100.$$

Here, I (Amp) is the average current generated, ∆t (sec) is the time period for which the experiment was run, while the ∆COD is the COD change from the initial value to the final value, V_an_ (L) is the wastewater volume which has been treated in the anodic chamber, number 8 in the equation is the constant against M.W. of the oxygen molecule (32 g/mol), and four electrons swapped per reduced oxygen molecule. Electrochemical experiments were repeated atleast twice (n = 2).

## Results and Discussions

### Iron Nanoparticle Characterization and CF-Modified Anode

**FIGURE 1:**
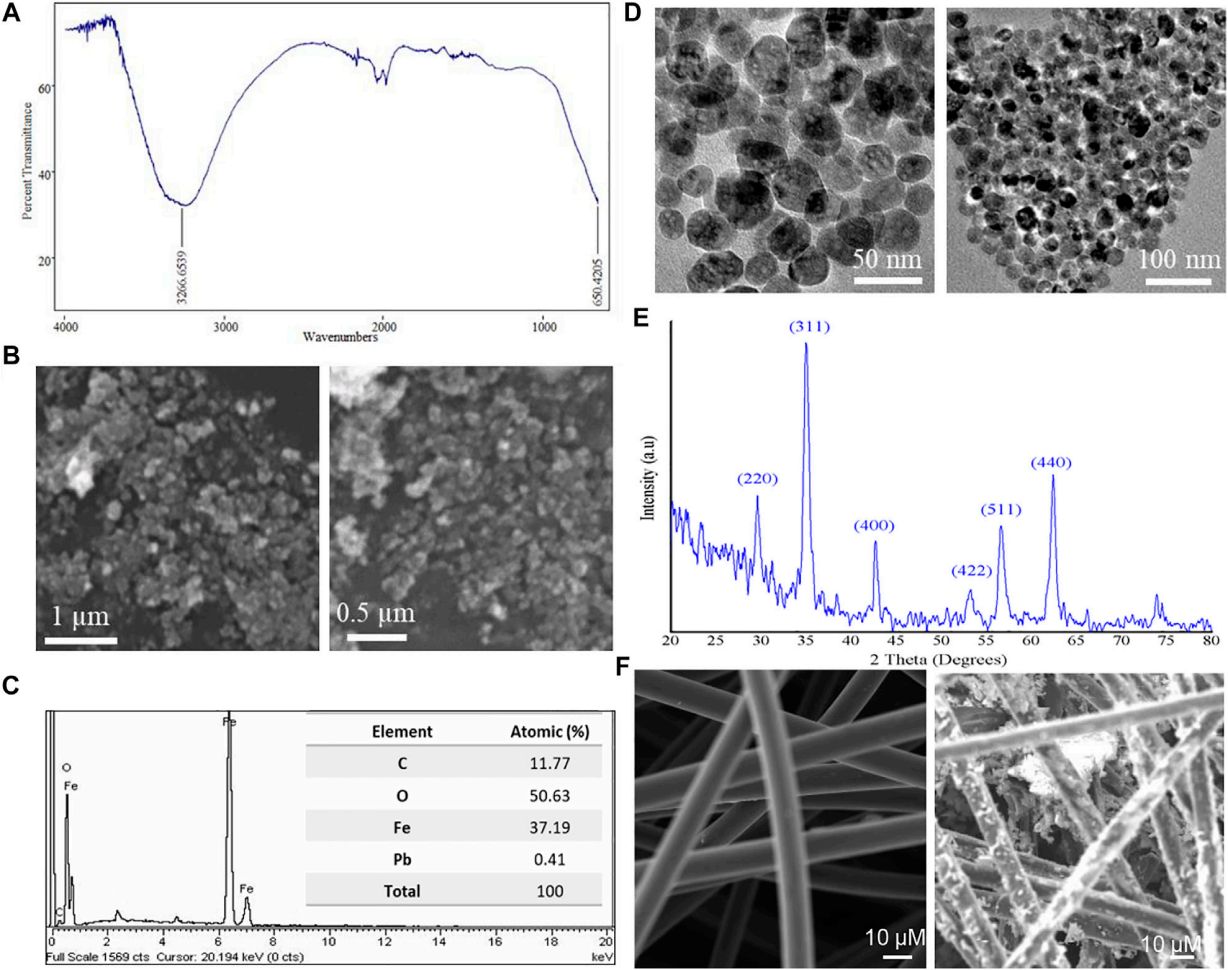
Fe_3_O_4_ nanoparticle characterization and the CF-modified anode. **(A)** FT-IR spectrum of synthesized nanoparticles in the range of 4,000–600 cm^−1^. **(B)** SEM images of synthesized nanoparticles. **(C)** EDX for elemental analysis of synthesized nanoparticles, showing the atomic percentage of elements present. **(D)** TEM images of synthesized nanoparticles. **(E)** XRD patterns of Fe NPs. **(F)** SEM images of bare CF and iron particle–modified CF.

First, the characterization of synthesized Fe NPs was performed. The FT-IR spectrum of synthesized nanoparticles was obtained in the range of 4,000–600 cm^−1^ to elucidate the formation of the nanomaterial and its purity. Figure 1A shows the FT-IR spectrum of the subjected nanoparticles. Two main peaks at 3,266 cm^−1^ and at 650 cm^−1^ are obtained which corresponds to the -OH bond stretching vibration and Fe–O bond vibrations, respectively. The FT-IR spectrum of the synthesized nanoparticles was in alignment with previously reported studies (Mishra et al., 2014; Touqeer et al., 2020). The morphology and surface of the synthesized nanoparticles was elucidated by SEM, as shown in Figure 1B. SEM images at different magnification levels exhibit that nanoparticles of very small size and uniform spherical morphology have been produced through a hydrothermal process. Few clumps/aggregations were also observed in the SEM images which might be due the magnetic character of Fe_3_O_4_ nanoparticles. Since the purity and morphology of the nanomaterial are crucial factors which could affect the cell performance, the elemental analysis of synthesized nanoparticles was done through the EDX. The purity and percentage composition of elements were obtained by the EDX plot, which is presented in Figure 1C. The atomic percentage of nanoparticles obtained by EDX is given in the table of Figure 1C. The ratio between atomic percentages of iron and O_2_ is similar to the atomic ratio of iron and O_2_ in Fe_3_O_4_, which affirms the formation of Fe_3_O_4_ nanoparticles. The morphology of nanoparticles was also determined by TEM using an accelerating voltage of 200 kV. From Figure 1D at different magnifications, one can clearly see a homogeneous morphology of Fe_3_O_4_ nanoparticles. The particles were somehow agglomerated due to the magnetic behavior of the nanoparticles. Furthermore, nanoparticles were spherical in nature with the appearance of mesopores in them which can be seen from the TEM images. The crystallinity and phase angle of the nanoparticles were estimated by XRD. The XRD plot of the synthesized nanoparticles is in good agreement with magnetite JPCDS Card No 019–0629. The obtained two theta values attribute to the hkl values of (220), (311), (400), (422), 511), and (440), respectively (Figure 1E). The sharp peaks and absence of any extraneous peaks affirms the purity and high crystallinity of the subjected nanoparticles, respectively. The crystal shape corresponding to the identified peaks (JPCDS Card No 019–0629) is a cubic spinel shape. The results obtained by XRD analysis match with that of previously reported results by other researchers (Xing et al., 2021). Once the nanoparticle synthesis was confirmed, it was coated on CF using the dip-and-dry method. Since surface properties of electrodes can substantially influence the operation of MFCs, especially bacterial growth and its adhesion to the electrodes (Yu et al., 2017), SEM analysis was conducted to confirm the morphology of the modified anode surface (Figure 1F). The images produced by SEM clearly revealed very smooth and clean fibers in the unmodified CF electrode. Conversely, a much irregular and rougher fiber surface in the modified electrode was observed, indicating that the presence of iron particles on the CF structures (Fe_3_O_4_@CF anode) promotes roughness on the CF electrodes. It is well-known that a rough surface assists additional microbial adhesion/colonization and ultimately enhanced electron transfer (Quan et al., 2015).

### Bioelectricity With Electroactive SRB and an Fe_3_O_4_@CF Electrode

**FIGURE 2:**
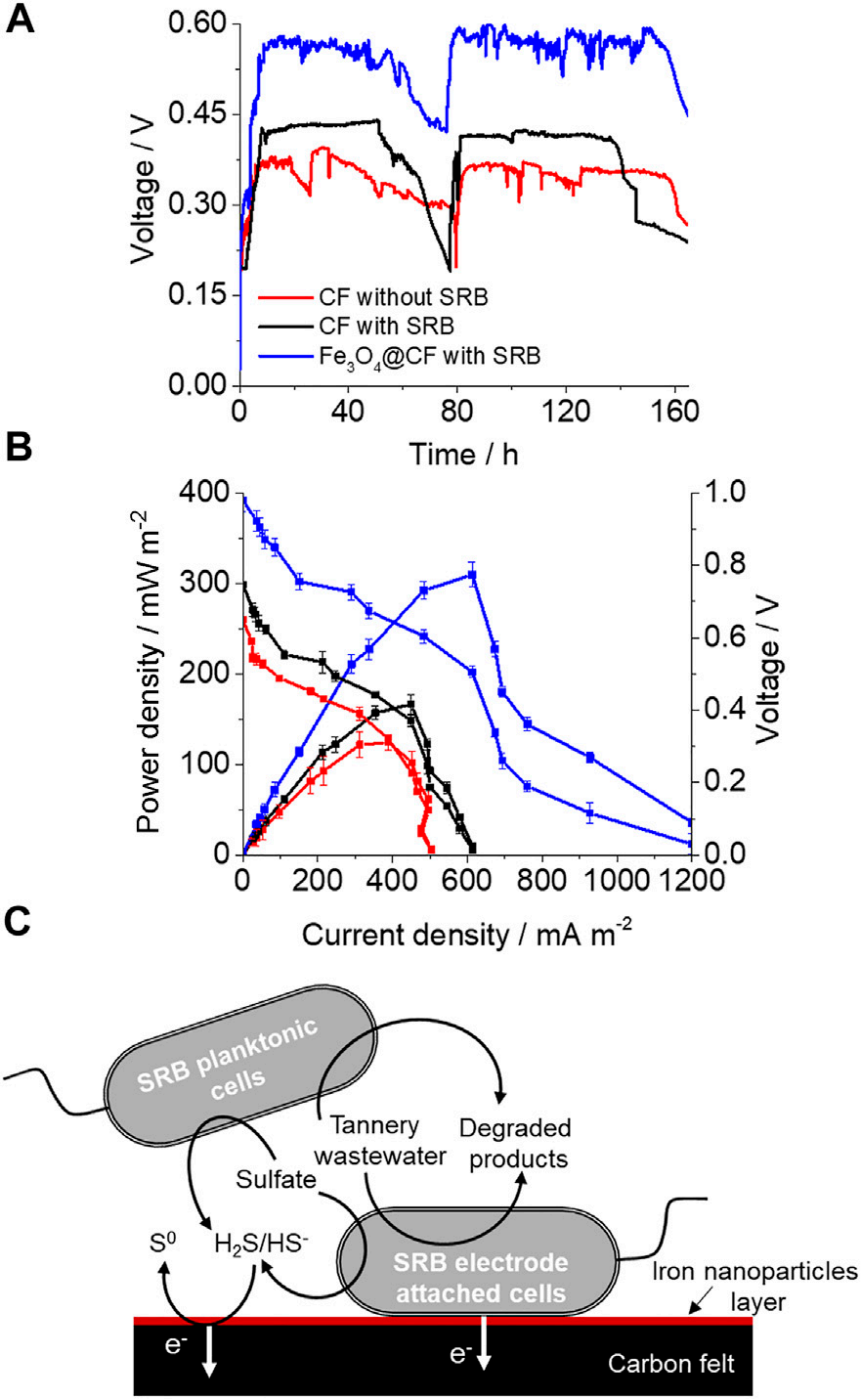
Voltage generation, power density curves, and predicted pathway involved in current generation. **(A)** Voltage generation by tannery wastewater of 500 mg/L COD strength for different MFC systems. **(B)** Polarization and power density curves for different electrode and culture conditions obtained by varying circuit resistance. **(C)** Proposed mechanism for simultaneous bioelectricity generation, tannery wastewater degradation, and sulfate reduction.

Voltage production by MFCs with the non–SRB-enriched CF electrode, SRB-enriched CF electrode, and SRB-enriched Fe NP–modified CF electrode (Fe_3_O_4_@CF electrode) was compared. Non-enriched and SRB-enriched bacteria were added to the respective MFCs. The microbial attachment and development of the biofilm on the electrode surface of the anodic chamber took place, and relatively stable voltage was generated (at 500 Ω) in the fourth week of MFC operation with tannery wastewater as the electron donor (at COD/SO_4_^2−^ of 2.0) in all cases (Figure 2A). In the fed batches, once the electron donors had been substantially consumed, the voltage started to decline and then attained the stable state again after the addition of fresh anolyte containing tannery wastewater, nutrients, and sulfate. In comparison to SRB non-enriched culture CF (0.39 V), SRB-enriched CF produced almost 15% higher voltage (0.45 V) (Figure 2A), suggesting the presence of more electroactive bacteria. A further substantial increase in voltage production was observed in the case of the SRB-enriched Fe_3_O_4_@CF electrode that was 1.53 times greater than the control without SRB-enriched culture and CF only (Figure 1A). This strongly suggests that the Fe_3_O_4_@CF electrode promotes electron transfer to the anode likely due to higher conductivity of Fe_3_O_4_ which can be used as conduit by SRB-like biogenic iron compounds (Murugan et al., 2018). At a tannery wastewater strength of 500 mg/L (COD) and COD/SO_4_^2−^ ratio of 2.0, a maximum power density and corresponding current density achieved were 124 ± 8 mW/m^2^ and 388 ± 24 mA/m^2^, respectively, obtained from power and polarization curves (Figure 2B). The achieved CE percentage was 24.6%, and the R_int_ calculated on the basis of the slope curve technique was 330 Ω. In comparison, while a maximum power density and corresponding current density achieved with SRB-enriched CF were 166 ± 11 mW/m^2^ and 449 ± 24 mA/m^2^, respectively, they were 309 ± 13 mW/m^2^ and 612 ± 25 mA/m^2^, respectively, for the SRB-enriched Fe_3_O_4_@CF electrode, clearly indicating the significance of electrode modification for enhancing maximum power density. Moreover, the enhanced power output suggested better bacterial growth and attachment on the Fe_3_O_4_@CF anode. Such facts have been reported in previous studies, where current generation enhancement was correlated with the bacterial biofilm growth (Reguera et al., 2006). Since in the mixed microbial community, many different microbes can play different roles including organic degradation and direct or mediated transfer of electrons from microbes to the electrode surface, the advantage associated with the presence of SRB is to outcompete methanogens that result in the suppression of CH_4_ formation in SRB-enriched cultures (Dar et al., 2008), and hence, improved MFC performance can be achieved as more electrons are available for transfer to electrodes. While electrons are transferred to the anode either directly via electrogens by c-type cytochrome or electrically conductive pili (nanowires), or indirectly via redox mediators (Lovley and Nevin, 2011), the SRB group also comprised species having c-type cytochromes that aid in DET to the anode, excluding the requirements of an exterior mediator (Kang et al., 2014). Apparently, there is a disadvantage linked with SRBs as part of the electron produced in the MFC was consumed for sulfate reduction, but the presence of continuous electron donors in real wastewater systems can help surmount this drawback to a considerable extent. Given that sulfates are reduced to sulfides by SRBs in the MFC, sulfides can be further oxidized to elemental sulfur and eventually assist in electricity production. Sulfide oxidation for current generation has already been well-investigated while treating sulfate-/sulfide-rich wastewater using SRBs (Lee et al., 2014). The soluble mediator-like sulfides allow all cells to contribute to current generation whether they are located in the biofilm or in the planktonic phase, and hence, planktonic cells may also play an important role in overall current generation. Based on the results, main possible steps encompassed in electricity generation, tannery wastewater biodegradation, and sulfate removal in this wotk are shown in Figure 2C. A comparison of MFC performance for different anode modified electrodes is also provided in Table 2.

**TABLE 2:** Comparison of MFC performance for different anode-modified electrodes.

Modified anode	Type of MFC	Methods for modification	Power density (mW/m^2^)	Inoculum source	Energy source	References
Reduced graphene oxide/MnO2 composite/carbon felt	Two-chamber	Electrode position	2065	Pre-acclimatized microbes from other reactors	Acetate	Zhang et al. (2016)
Polyaniline (PANI)/CF	Two-chamber	*In situ* polymerization	27.3	Anaerobic	Acetate	Li et al. (2011)
Tartaric acid–doped PANI/carbon cloth	Two-chamber	*In situ* polymerization	490	*S. oneidensis*	Lactate	Liao et al. (2015)
Graphene/PANI/carbon cloth	Two-chamber	Electrochemical	1,390	Anaerobic sludge	Acetate	Hou et al. (2013)
PANI–carbon nanotubes/graphite felt	Two-chamber	Electropolymerization	257	*S. putrefaciens*	Acetate	Cui et al. (2015)
Carbon nanotubes/PANI/nickel foams	Single-chamber	Mechanical coating	42	*E. coli*	Glucose	Qiao et al. (2007)
Polypyrrole (PPy)/anthraquinone-2,6-disulfonic disodium salt/carbon felt	Two-chamber	Electropolymerization	1,300	*S. decolorationis*	Lactic acid	Feng et al. (2010)
PPy/carbon nanotubes/MnO_2_	Two-chamber	Electropolymerization	1,125.4	Mixed sewage culture	Sewage wastewater	Mishra and Jain, (2016)
Iron oxide/carbon felt	Two-chamber	Deposition coating	309	Mixed SRB	Tannery wastewater	This study

### Tannery Wastewater Treatment Performance

**FIGURE 3:**
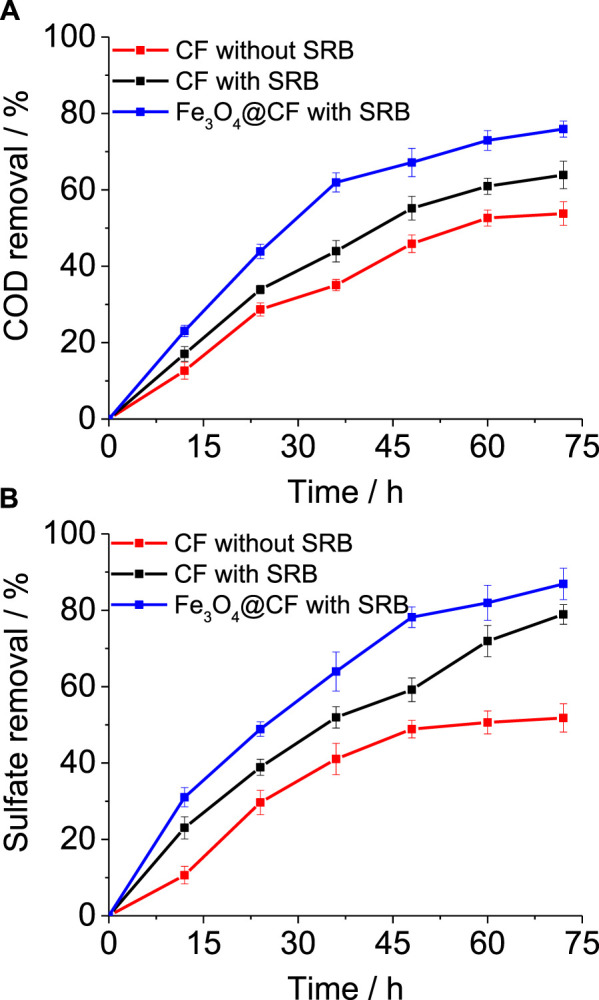
COD and sulfate removal profile with different electrodes and culture conditions. **(A)** COD and **(B)** sulfate removal efficiency comparison in MFCs for the non–SRB-enriched CF electrode, SRB-enriched CF electrode, and SRB-enriched Fe3O4@CF electrode.

MFC performance in relation to COD removal and sulfate reduction was evaluated in all three MFC systems. A consistent initial COD and sulfate concentration were maintained at 500 mg/L and 250 mg/L, respectively, in all these MFCs. The removal percentage was observed for 72 h by taking samples after intervals of 12 h. A 53 ± 3.1% COD removal was achieved in the current study with non–SRB-enriched CF (Figure 3A). In comparison, SRB-enriched CF showed 10% higher COD removal (63 ± 3.6%), indicating the importance of SRB in degrading complex compounds in tannery wastewater. Nevertheless, COD removal was further enhanced with the SRB-enriched Fe_3_O_4_@CF electrode where 75 ± 2.1% COD removal was accomplished (Figure 3A). The accelerated biodegradation of tannery wastewater in the MFCs with SRB enriched-Fe_3_O_4_@CF can be attributed to the anode electrode which provides a pathway for electron transfer to the cathode via an external circuit that can eventually boost the metabolic rate of microbes in the presence of these non-exhaustible electron acceptors (Mohanakrishna et al., 2020; Hu et al., 2018). Furthermore, the improvement in tannery wastewater biodegradation rates attributed to SRB cultures suggests the enrichment of microbes which were highly specific to the biodegradation of recalcitrant pollutants in tannery wastewater. SRB cultures performing more specific and efficient functions in anaerobic systems have already been shown in previous studies. In terms of sulfate reduction, a similar trend was observed as with COD removal. Highest sulfate removal was demonstrated by the MFC with SRB-enriched Fe_3_O_4_@CF (88 ± 4.1%), followed by SRB-enriched CF (78 ± 2.6%) and non–SRB-enriched CF (51 ± 3.7%) (Figure 3B). While theoretical calculations showed that the COD/SO_4_^2-^ ratio of 0.67 is sufficient for completely removing the organics (as COD) by SRBs during sulfate reduction (Rasool et al., 2015), it was reported earlier that in real systems, a COD/SO_4_^2-^ ratio of 1.7–2.5 is optimum for microbial growth while reducing sulfates in fed batches in the presence of SRBs (Miran et al., 2018). Therefore, in this study, an optimal concentration of COD/SO_4_^2-^ ratio of 2.0 was selected to avoid any limitations as a low COD/SO_4_^2-^ ratio can result in inferior MFC performance, as reported by Ghangrekar et al. (Ghangrekar et al., 2010). Furthermore, good repeatability of MFC cycles in this work indicates the robustness of electroactive SRB for COD and sulfate removal.

## Conclusion

Fe NP–modified CF with mixed SRB cultures can be utilized in MFCs for tannery wastewater treatment with simultaneous sulfate removal and electricity production. An effective tannery wastewater treatment was demonstrated that is beneficial for resolving environmental and local societal issues. Although power output with the modified electrode and SRBs improved substantially, the tannery wastewater biodegradation rate can be further enhanced by quantifying the type of microbial culture present and further fine-tuning their relative abundance. For commercial purposes, there is a requirement of further modification and optimization of MFC designs and operation. For instance, a continuous system is required to be developed and optimized for industrially acceptable performance.

## Data Availability

The raw data supporting the conclusion of this article will be made available by the authors, without undue reservation.
